# Association between incarceration and incident cardiovascular disease events: results from the CARDIA cohort study

**DOI:** 10.1186/s12889-021-10237-6

**Published:** 2021-01-26

**Authors:** Jordan Coleman, Donald M. Lloyd-Jones, Hongyan Ning, Norrina B. Allen, Catarina I. Kiefe, Emily A. Wang, Mark D. Huffman

**Affiliations:** 1 Department of Preventive Medicine, Northwestern University Feinberg School of Medicine, 680 N, 710 N Lake Shore Drive, Suite 800, Chicago, IL 60611 USA; 2 University of Massachusetts Medical School, 368 Plantation Street, AS7-1077, Worcester, MA 01605 USA; 3 Department of Internal Medicine, Yale School of Medicine, PO Box 208056, 333 Cedar Street, New Haven, CT 06520 USA; 4 The George Institute for Global Health, 1 King Street, 5th floor, Sydney, NSW 2042 Australia

**Keywords:** Incarceration, Cardiovascular disease, Epidemiology

## Abstract

**Background:**

Incarceration has been associated with higher cardiovascular risk, yet data evaluating its association with cardiovascular disease events are limited. The study objective was to evaluate the association between incarceration and incident fatal and non-fatal cardiovascular disease (CVD) events.

**Methods:**

Black and white adults from the community-based Coronary Artery Risk Development in Young Adult (CARDIA) study (baseline 1985–86, *n* = 5105) were followed through August 2017. Self-reported incarceration was measured at baseline (1985–1986) and Year 2 (1987–1988), and fatal and non-fatal cardiovascular disease events, including coronary heart disease, stroke, and heart failure, and all-cause mortality, were captured through 2017. Analyses were completed in September 2019. Cumulative CVD incidence rates and Cox proportional hazards were compared overall by incarceration status. An interaction between incarceration and race was identified, so results were also analyzed by sex-race groups.

**Results:**

351 (6.9%) CARDIA participants reported a history of incarceration. Over 29.0 years mean follow-up, CVD incidence rate was 3.52 per 1000 person-years in participants with a history of incarceration versus 2.12 per 1000 person-years in participants without a history of incarceration (adjusted HR = 1.33 [95% CI, 0.90–1.95]). Among white men, incarceration was associated with higher risk of incident cardiovascular disease (adjusted HR = 3.35 [95% CI, 1.54–7.29) and all-cause mortality (adjusted HR = 2.52 [95% CI, 1.32–4.83]), but these associations were not statistically significant among other sex-race groups after adjustment.

**Conclusions:**

Incarceration was associated with incident cardiovascular disease rates, but associations were only significant in one sex-race group after multivariable adjustment.

**Supplementary Information:**

The online version contains supplementary material available at 10.1186/s12889-021-10237-6.

## Background

The United States has the world’s highest incarceration rate, and incarceration rates have increased dramatically over the past 30 years, especially among some sex-race/ethnic groups [1]. For example, the lifetime risk for incarceration is estimated to be 1 in 3 among Black men and 1 in 6 among Hispanic/Latino men [2]. Cardiovascular disease (CVD) is the leading cause of death among the estimated 2.2 million individuals who are incarcerated in the United States [3]. Following release from incarceration, CVDs are the leading causes of hospitalization among those recently incarcerated [4]. Longitudinal community-based cohort studies have demonstrated an association between incarceration and risk factors for CVD, including incident hypertension, as well as corresponding cardiac structural changes [5]. Mechanisms relating incarceration to CVD have been proposed by a National Heart, Lung, and Blood Institute workshop committee, including socio-demographic factors associated with disproportionate CVD risk, stress and mental health associated with incarceration, potentially unhealthy behaviors and limited health care during incarceration leading to increased burden of CVD risk factors, and poorer health care access following release [3]. However, to our knowledge, longitudinal, community-based cohort data exploring the relationship between incarceration and incident CVD events have not been published. Therefore, the objective of this analysis was to evaluate the long-term association between adult incarceration history and incident fatal and non-fatal CVD events among white and Black participants in the Coronary Artery Risk Development in Young Adults community-based cohort study.

## Methods

### Sample

The Coronary Artery Risk Development in Young Adults (CARDIA) cohort study enrolled 5115 black and white men and women between 18 and 30 years old and who were free from CVD at baseline in 1985–1986 [6]. Participants were recruited from Chicago, Illinois; Birmingham, Alabama; Oakland, California; and Minneapolis, Minnesota. The sampling methods resulted in a baseline cohort balanced in terms of age, sex, race, and education. Investigators have re-examined participants at in-person examinations at years 2, 5, 7, 10, 15, 20, 25, and 30 (2015–16). Retention rates among surviving participants at each in-person examination were 91, 86, 81, 79, 74, 72, 72, and 71%, respectively. Contact is maintained with participants via telephone, mail, or email every 6 months, with annual, interim medical history ascertainment. Over the last 5 years, > 90% of surviving cohort members have been directly contacted, and follow-up for vital status is virtually complete through related contacts and intermittent National Death Index searches. All CARDIA participants included in this study provided informed consent. The CARDIA study has been reviewed and approved by the Institutional Review Boards of all participating institutions.

Among 5115 CARDIA participants, we excluded 6 participants with cardiovascular disease events at or prior to Exam 2, 2 participants who were transgender, 1 participant who withdrew consent, and 1 participant without a response to incarceration history. We included 5105 participants in the analysis (Supplemental Figure).

### Ascertainment of incarceration

CARDIA participants were assessed on their history of incarceration at two time points. At baseline and Year 2 examinations, participants were asked, “During the past year, did any of the following happen to you?” with “Went to jail” one of the options. The Year 2 follow-up examination allowed individuals to report any incarceration event that happened since baseline, although no similar questions were asked at subsequent exams. Recorded responses to these two questions allowed for the creation of an incarceration exposure variable to account for any reported time spent in jail (generally considered < 1 year of incarceration) or prison (1 year or greater period of incarceration) from 1 year prior to the study through the Year 2 examination. Among participants who reported more than 1 incarceration event, the earliest incarceration date was selected as the exposure of interest. No reasons for incarceration were captured.

### Measurement of cardiovascular disease risk factors

Trained study personnel captured socio-demographic, behavioral, and CVD risk factor data at each exam with quality control protocols in place. Age, sex, race/ethnicity, highest education level, and annual family income were self-reported. Baseline annual family income below $24,999 per year was defined as low socioeconomic position by meeting the 250% federal poverty line in 1985 [7].

Smoking history was assessed using a questionnaire administered by interviewers. Heavy alcohol intake was defined using the National Institute on Alcohol Abuse and Alcoholism thresholds (> 14 drinks/week for men; or > 7 drinks/week for women) [8]. Information about illicit drug use was obtained through interview-style questionnaires that asked specifically about use of cocaine and amphetamines. Participants were considered current users if they had used in the past month. Physical activity information was collected using an interviewer-administrated CARDIA Physical Activity History questionnaire that asked about 13 specific activities over the course of the prior year [9].

CVD risk factors were recorded at baseline and at subsequent exams. For measurement of body mass index, trained and certified technicians weighed participants using a standard balance beam scale while wearing light clothing. A wall-mounted stadiometer was used for height measurements, which were recorded to the nearest half centimeter. Participants’ blood pressures were recorded using a random zero sphygmomanometer. Both systolic and diastolic blood pressures were recorded three times at an interval of 1 minute, and the mean of the last 2 measures was recorded. Fasting serum total cholesterol and high-density lipoprotein cholesterol levels were measured enzymatically from plasma venipuncture samples stored at -70 °C. The Friedewald equation was used to calculate low-density lipoprotein cholesterol levels. Fasting plasma glucose levels were also measured from stored samples. Data were captured about medical history and blood pressure, cholesterol, and diabetes medications.

### Outcome ascertainment

Ascertainment of fatal and non-fatal CVD events, including coronary heart disease, stroke, and heart failure, and all-cause mortality was performed annually between baseline to the present through telephonic follow-up, in-person examinations, and review of National Death Index database (details at: http://www.cardia.dopm.uab.edu). Hospital records were sought for self-reported outcomes, and central adjudication was performed by trained physicians to ascertain CVD events. The last date of follow-up for this analysis was August 31, 2017.

### Statistical analysis

CARDIA participants with and without a self-reported history of adult incarceration exposure were compared in terms of socio-demographics, behaviors, medical history, and CVD risk factors. Cumulative composite CVD event incidence rates, including fatal and non-fatal coronary heart disease, stroke, and heart failure, and all-cause mortality were assessed beginning after the Year 2 examination to capture exposure data prior to any events. We also explored the association between incarceration and coronary heart disease, stroke, and heart failure separately. We created unadjusted and adjusted Cox proportional hazards models after confirming that the proportionality assumption was met. Participants who died from non-cardiovascular causes were censored from the analyses at their date of death. We first adjusted for baseline age, sex, race/ethnicity and then adjusted for baseline education, smoking, excessive alcohol consumption, physical activity, body mass index, systolic blood pressure, use of blood pressure lowering medication, total and high-density lipoprotein cholesterol, and field center. Variance Inflation Factors were calculated to diagnose collinearity between multiple variables and were well below values indicative of multicollinearity (range: 1.04–1.37). We report the results of the complete case analysis as the primary analysis due to the low rate of missingness (0.2%) of these exposure data. As part of our standard analyses of the models, we identified an interaction between incarceration and race with incident cardiovascular disease by calculating the relative excess risk for interaction, which was significant on both additive and multiplicative scales. We observed the significant interaction term of race and incarceration, and therefore, report these exploratory, post hoc results by sex-race strata for incident cardiovascular disease and all-cause mortality. There were too few events in subtypes of cardiovascular disease events among some sex-race groups to create stable model estimates so we do not report these results. We also performed post hoc sensitivity analyses using competing Cox models to account for the potential influence of competing risk of death due to non-CVD causes since risk of death from non-CVD causes may vary by incarceration status [10]. SAS v 9.4 (Cary, NC) was used for statistical analyses. Analyses were completed in September 2019. A two-sided *P* value < 0.05 was used to define statistical significance.

### Role of funding source

The sponsor did not participate in the current study design; in the collection, analysis, and interpretation of data; in the writing of the report; and in the decision to submit the paper for publication.

## Results

### Participants

Among the eligible sample (5114 participants; 1 participant rescinded consent for participation), we included 5105 participants (99.8%) in our analyses because 9 participants either experienced an event, were lost to follow-up, or underwent gender reassignment between the baseline examination and Year 2 follow-up.

### Adult incarceration

Of the 351 (6.9%) CARDIA participants who reported a history of incarceration, 155 (44%) reported having been in jail in the year prior to enrolling in the study, and 136 (39%) responded yes to the question at the Year 2 examination, indicating they had been incarcerated between the start of the study and Year 2 (Table S1). Sixty (17%) participants responded yes to the question on both forms, identifying multiple incarceration exposures.

Table 1 shows the baseline characteristics of the sample, stratified by incarceration history. Compared with participants without a history of incarceration, participants with a history of incarceration were significantly younger and more likely to be male, Black, have a lower level of education and have an annual family income less than $25,000 at baseline. Participants with a history of incarceration were also more likely to smoke at baseline, have heavy alcohol use, report illicit drug use, and have a higher baseline systolic blood pressure. Serum low-density lipoprotein and high-density lipoprotein cholesterol and fasting plasma glucose levels were similar between groups.

**Table 1:** Baseline Characteristics According to Incarceration History Status, CARDIA Study: 1985–1988

Baseline Characteristic	Incarceration (***n*** = 351)	No Incarceration History (***n*** = 4754)	***P*** Value
Age, mean (SD), years	24.0 (3.7)	24.9 (3.6)	<.0001
Male sex, No. (%)	268 (76.4)	2054 (43.2)	<.0001
Black race, No. (%)	254 (72.4)	2379 (50.0)	<.0001
Education, mean (SD), years	12.4 (1.8)	13.9 (2.3)	<.0001
Individual annual income <$25,000, No. (%)^a^	173 (61.8)	1462 (36.7)	<.0001
Smoker, No. (%)	190 (55.1)	1351 (28.6)	<.0001
Heavy alcohol use, No. (%)	98 (28.0)	550 (11.6)	<.0001
Illicit drug use, No. (%)^b^	86 (26.5)	312 (7.3)	<.0001
Heavy or moderate exercise, median (IQR), MET-minutes per week	443 (254, 714)	356 (192, 571)	< 0.001
Body mass index, mean (SD), kg/m^2^	24.1 (4.3)	24.5 (5.1)	0.11
Systolic blood pressure, mean (SD), mmHg	113.1 (11.4)	110.2 (10.9)	<.0001
Diastolic blood pressure, mean (SD), mmHg	69.0 (10.7)	68.6 (9.5)	0.38
Blood pressure medication, No. (%)	8 (2.3)	107 (2.3)	0.85
Total cholesterol, mean (SD), mg/dl	175.4 (34.9)	176.9 (33.4)	0.43
Low density lipoprotein cholesterol, mean (SD), mg/dl	106.1 (32.1)	109.3 (31.1)	0.06
High density lipoprotein cholesterol, mean (SD), mg/dl	54.1 (14.7)	53.1 (13.1)	0.17
Triglycerides, median (IQR), mg/dl	65 (47.5, 90.5)	62 (45, 84)	0.04
Fasting plasma glucose, mean (SD), mg/dl	83.4 (9.4)	82.5 (16.7)	0.36
Diabetes medication, No. (%)	1 (0.3)	13 (0.3)	1.00

*SD* standard deviation

*IQR* interquartile range

*MET* metabolic equivalent

^a^836 (16%) participants did not respond to this question

^b^532 (10%) participants did not respond to this question

### Primary analysis

**Fig. 1:**
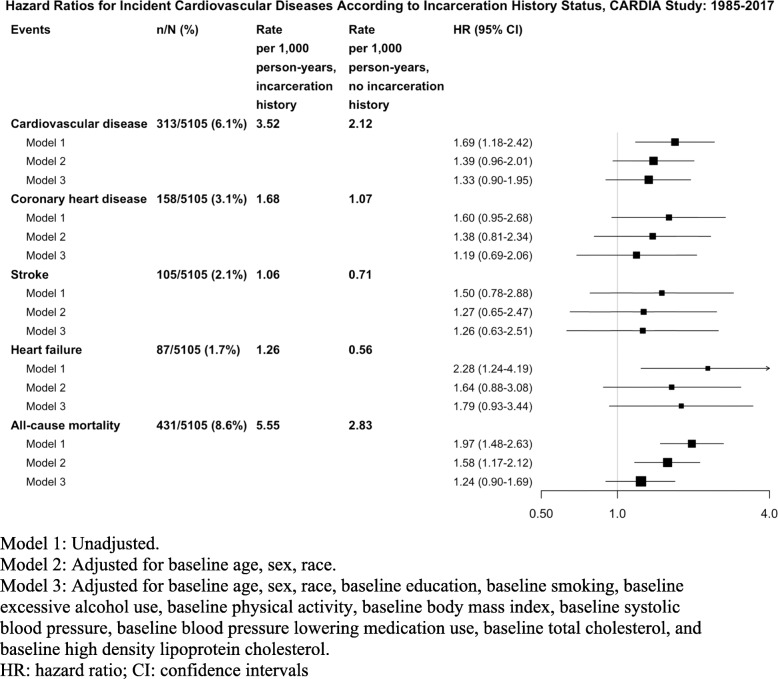
Hazard Ratios for Incident Fatal and Non-fatal Cardiovascular Diseases According to Incarceration History Status, CARDIA Study: 1985–2017

Over mean follow-up of 29.0 years, there were 313 cases of incident CVD events identified: 33 among participants with a history of incarceration, and 280 among participants without a history of incarceration (9.4% versus 5.9%, *P* = 0.008, Table S2). The most common event was incident coronary heart disease among both groups. The incidence rate for CVD was 3.52 per 1000 person-years in participants with a history of incarceration compared with 2.12 per 1000 person-years (*p* = 0.03) in participants without a history of incarceration (unadjusted hazard ratio, HR = 1.69; 95% confidence intervals [CI] 1.18–2.42; Fig. 1).

After adjustment, the association was no longer statistically significant (adjusted HR = 1.33 [95% CI, 0.90–1.95]) in the overall cohort. The direction and magnitude of findings were similar for all-cause mortality. The association between incarceration and incident CVD was also similar after accounting for the competing risk of non-CVD death (adjusted HR = 1.28 [95% CI, 0.86–1.91], Table S3).

### Secondary analyses

We stratified analyses by sex and race after identifying an interaction between incarceration and race with incident CVD, and report event rates by sex-race group in Table 2. Baseline characteristics according to sex-race group and incarceration status are reported in Table S4. Among white men, participants with a history of incarceration experienced a higher rate of incident CVD compared with those without a history of incarceration (13.7% versus 6.22%, *P* = 0.01); this finding was not observed among Black men (8.2% versus 8.9%, *P* = 0.74), who were at the overall highest risk for CVD, nor among women of either race. The incidence rate for CVD was 5.20 per 1000 person-years among white men with a history of incarceration compared with 2.25 per 1000 person-years among white men without a history of incarceration (adjusted HR = 3.35 [95% CI, 1.54–7.29], Table 3). Among other sex-race groups with a history of incarceration, there was no statistically significant association with incident CVD (Black men, adjusted HR = 0.82 [95% CI, 0.44–1.52]; white women, adjusted HR = 1.60 [95% CI, 0.20–12.57]; Black women, adjusted HR = 1.22 [95% CI, 0.44–3.40]. Incarceration was also associated with all-cause mortality in white men (adjusted HR = 2.52 [95% CI, 1.32–4.83]) but not among other sex-race groups.

**Table 2:** Event Rates for Incident Fatal and Non-fatal Cardiovascular Diseases and All-Cause Mortality According to Incarceration History Status Stratified by Sex and Race, CARDIA Study

Event	White Men		***P*** Value	Black Men		***P*** Value	White Women		***P*** Value	Black Women		***P*** Value
	Incarceration History (***n*** = 73)	No Incarceration History (***n*** = 1094)		Incarceration History (***n*** = 195)	No Incarceration History (***n*** = 960)		Incarceration History (***n*** = 24)	No Incarceration History (***n*** = 1281)		Incarceration History (***n*** = 59)	Incarceration History (***n*** = 1419)	
Cardiovascular disease, No.^a^ (%)	10 (13.7)	68 (6.2)	0.01	16 (8.2)	86 (9.0)	0.73	1 (4.2)	37 (2.9)	0.71	6 (10.2)	89 (6.3)	0.23
Coronary heart disease, No. (%)	7 (9.6)	56 (5.1)	0.10	7 (3.6)	39 (4.1)	0.76	0 (0.0)	21 (1.6)	0.53	2 (3.4)	26 (1.8)	0.39
Stroke, No. (%)	1 (1.4)	9 (0.8)	0.62	5 (2.6)	30 (3.1)	0.68	0 (0.0)	12 (0.9)	0.63	4 (6.8)	44 (3.1)	0.12
Heart failure, No. (%)	2 (2.7)	7 (0.5)	0.05	7 (3.6)	32 (3.3)	0.85	1 (4.2)	5 (0.4)	<.01	2 (3.4)	31 (2.2)	0.53
All-cause mortality, No. (%)	13 (17.8)	78 (7.1)	<.01	32 (16.4)	135 (14.1)	0.40	1 (4.2)	63 (4.9)	0.87	7 (11.9)	102 (7.2)	0.18

^a^Some participants had more than one event, which explains why the individual outcomes are greater than the total number of cardiovascular disease events

**Table 3:** Hazard Ratios for Incident Fatal and Non-fatal Cardiovascular Diseases and All-Cause Mortality According to Incarceration History Status, Stratified by Sex and Race, CARDIA Study

Event	n/N	Rate per 1000 person-years, incarceration history	Rate per 1000 person-years, no incarceration history	Model 1 HR (95% CI)	Model 2 HR (95% CI)	Model 3 HR (95% CI)
**Cardiovascular diseases**						
White Men	78/1167	5.20	2.25	2.40 (1.23–4.66)	2.87 (1.48–5.62)	3.35 (1.54–7.29)
Black Men	102/1155	3.13	3.33	0.94 (0.55–1.61)	1.01 (0.59–1.73)	0.82 (0.44–1.52)
White Women	38/1305	1.44	1.02	1.40 (0.19–10.18)	1.45 (0.20–10.57)	1.60 (0.20–12.57)
Black Women	95/1478	3.66	1.20	1.63 (0.71–3.72)	1.66 (0.73–3.80)	1.22 (0.44–3.40)
**All-cause mortality**						
White Men	91/1167	6.53	2.54	2.60 (1.44–4.67)	2.89 (1.60–5.22)	2.52 (1.32–4.83)
Black Men	167/1155	6.15	5.14	1.23 (0.83–1.80)	1.29 (0.88–1.90)	0.92 (0.55–1.52)
White Women	64/1305	1.44	1.72	0.83 (0.12–6.00)	0.87 (0.12–6.24)	0.63 (0.08–4.87)
Black Women	109/1478	4.19	2.54	1.49 (0.69–3.23)	1.54 (0.71–3.35)	1.24 (0.49–3.13)

Model 1: Unadjusted

Model 2: Adjusted for baseline age

Model 3: Adjusted for baseline age, sex, race, baseline education, baseline smoking, baseline excessive alcohol use, baseline physical activity, baseline body mass index, baseline systolic blood pressure, baseline blood pressure lowering medication use, baseline total cholesterol, and baseline high density lipoprotein cholesterol

*HR* hazard ratio, *CI* confidence intervals

## Discussion

### Summary of results

We demonstrated a higher rate of incident CVD over a 29-year period of follow-up among individuals with a history of incarceration in a community-based cohort, although this finding was no longer statistically significant after multivariable adjustment in most sex-race groups. Results were similar for all-cause mortality. In post hoc analyses, the association between incarceration and incident CVD and all-cause mortality was evident among white men, but not among other sex-race groups.

Mechanisms of the proposed relationship relate to individual-, family- and community-level factors before, during, and after incarceration [3]. First, individuals, families, and communities with lower socioeconomic position have higher levels of CVD and common CVD risk factors because of fewer “flexible resources” for healthier living based on the fundamental cause theory [11]. Lower socioeconomic position groups also have disproportionate rates of incarceration, which can exacerbate stress, depression, and mental illness. While incarcerated, individuals face variability in the availability and quality of clinical care available, sedentarism and unhealthy diets that can worsen CVD risk factors, and limited autonomy and self-efficacy to manage health conditions. Following release, previously-incarcerated individuals often return to resource-limited communities and face further socioeconomic disadvantage because of their history of incarceration, based on policies that may limit job and educational grant opportunities [3].

The observed attenuation of the unadjusted association and sex-race differences may be driven by several factors. First, while it is possible that no association exists, the small number of events among individuals with a prior history of incarceration suggests that this relationship may be underpowered to detect such an overall association after adjustment in all sex-race groups. The hypothesized relationship may be mediated through CVD risk factors included in the multivariable models, which explains the attenuation after adjustment. For example, a 2020 systematic review of 26 longitudinal studies demonstrated that incarcerated individuals were gained, on average, 5.3 kg (95% CI: 0.5–10.1 kg) in weight during their period of incarceration, though the trends in other risk factors were less clear [12]. Second, losses to follow-up among Black men were higher than among other sex-race groups, and up to 65% of those losses at later examinations may have been due to incarceration [13], which limits the ability to capture non-fatal cardiovascular disease events occurring during incarceration, including among those with a history of incarceration. Third, other sociodemographic factors, including poverty, stress, and social disadvantage may be collinear with race/ethnicity and attenuate an association, though we adjusted for education as a marker for socioeconomic position. However, these relationships are complex and difficult to identify the independent contribution of each to the hypothesized relationship. Fourth, CVD event rates were higher among Black men and women, including in the unexposed groups. For example, compared with white men, Black men had a 48% higher rate of incident CVD. White men with a history of incarceration also had a higher risk for all-cause mortality compared with white men without incarceration, but this rate was 51% lower compared with the all-cause mortality rate among Black men. Fifth, ascertainment of the exposure did not differentiate between incarceration type or length, and there may be a differential pattern of association based on these exposure-level factors. Sixth, post-release neighborhood disadvantage may be relatively greater among former inmates who are white compared with other race/ethnic groups after controlling for pre-prison characteristics based on the National Longitudinal Survey of Youth [14]. Finally, there was likely some misclassification of incarceration status, because of under-reporting of incarceration due to social desirability bias or because some individuals may have been incarcerated > 1 year before the baseline examination or after the time of the Year 2 examination, or could not attend the Year 2 examination, any of which would have tended to bias our findings of an association between incarceration and incident CVD events toward the null, particularly among Black men.

### Comparison with prior studies

Previous research has demonstrated associations between prior incarceration and higher burden of CVD risk factors and subclinical CVD, including weight gain during incarceration [12] and incident hypertension and post-release hospitalization due to CVDs [4, 5]. For example, CARDIA data demonstrate a 1.7-fold higher odds of incident hypertension and a 2.7-fold higher odds of left ventricular hypertrophy among those with a history of incarceration [5]. Individuals with a history of incarceration also had a 2.5 higher odds of not having access to regular medical care. Among Medicare beneficiaries with a recent incarceration event, the odds of hospitalization were 2.5, 2.1, and 1.8 times higher at 7, 30, and 90 days post-release, respectively, compared with matched controls, including CVD hospitalization [4]. However, these individuals were more likely to be Medicare beneficiaries based on disability status or end-stage renal disease compared with the general Medicare population. A 15.5-year follow-up linkage study among 23,510 previously incarcerated individuals in Georgia (95% men, 67% Black) demonstrated a 1.77 (95% CI, 1.67–1.88) higher standardized mortality risk compared with non-incarcerated Georgians [15]. The authors estimated that 62% of the excess post-release risk was due to HIV, cancer, homicide, transportation-related injury, and accidental poisoning; heart disease rates were not higher among previously incarcerated individuals than expected, after adjustment. Higher rates have been reported, including a 3.5-fold (95% CI, 3.2–3.8) higher mortality rate observed among previously incarcerated individuals in Washington state, where a 2.1-fold (95%CI, 1.6%-2.1) higher CVD-related mortality rate was observed over a mean follow-up of 1.9 years [16].

Other research has reported a higher risk for self-reported obesity, CVD, and fair or poor health among women who have a family member incarcerated [17], demonstrating the broad influence of incarceration on family health. Neighborhood incarceration rates are associated with CVD risk factors, which suggests potential environmental drivers of health behaviors and factors prior to and following incarceration [18]. A 2018 systematic review of 11 studies summarized the role of interventions to improve health behaviors and factors among individuals during their incarceration, including group-based interventions to improve activity, nutrition, blood pressure, cholesterol, and smoking cessation [19]. While risk factor management during incarceration may help reduce long-term risks, residual risk factor exposure prior to incarceration, accumulation following release, and differential access to health care may influence long-term outcomes.

This study provides unique, community-based, longitudinal data reporting on the long-term relationship between incarceration and CVD events. Whether these associations are independent of risk factors that operate within the known causal pathway for incident CVD or also operate more broadly in terms of differences in early life exposures, social, environmental, or behavioral factors, and access to and quality of preventive and acute treatment is uncertain but plausible. At a minimum, our data demonstrate potential CVD-related hazards of incarceration and suggest the need for primordial and primary CVD prevention among incarcerated young adults, and further research to quantify risks more precisely and to identify successful prevention strategies.

### Strengths and limitations

This study has several strengths, including a recruiting a large bi-racial, community-based sample with rigorous 30 year follow-up. This study also has limitations, including limited number of events in two race/ethnic groups and thus power to detection a potential association, as well as risk of recall bias, leading to underreporting of previous incarceration, though this would likely attenuate any potential associations. While CARDIA used robust methods to capture events, some non-fatal CVD events may not have been captured, especially among Black men in whom losses to follow-up were disproportionately higher due to incarceration. Analyses aimed to adjust for confounders as well as risk factors that operate within the causal pathway, which may lead to over-adjustment. Analyses did not adjust for factors that may have influenced access to medical care, such as health insurance, which was not captured at baseline. We sought to minimize missingness of covariates in our models by selecting parsimonious models, but this may have influenced these results. This study sought to establish an association between incarceration and incident CVD, and the observed associations among white men may be due to chance given the post hoc nature of these analyses and should be interpreted with caution. We did not explore the potential influence of length or cause of incarceration, which may affect these results. We did not capture data on incarcerations earlier or later in adulthood, so not all incarceration exposures were captured, which may attenuate or abrogate a potential association. On the other hand, this potential misclassification could also bias the results of a potential association between incarceration and incident cardiovascular disease events toward the null. It remains uncertain if avoiding or reducing incarceration would lower the incidence of CVD, although there are other, arguably larger, individual and societal benefits to minimizing incarceration.

## Conclusions

A history of incarceration was associated with incident CVD event rates overall in a young, biracial community-based cohort study, but this relationship was significant after multivariable adjustment in some sex-race groups. These provocative data merit careful interpretation and replication in other studies to understand their full implications, and further research in larger cohorts or with longer follow up may improve precision of these estimates, and indicate potential reasons underlying the associations.

## Supplementary Information

**Additional file 1: Supplemental Figure.** Participant Flowchart

**Additional file 2: Supplemental Table 1.** Incarceration History by Exam Year, Stratified by Sex and Race, CARDIA Study. **Supplemental Table 2.** Unadjusted Event Rates for Incident Fatal and Non-fatal Cardiovascular Diseases and All-Cause Mortality According to Incarceration History Status, CARDIA Study: 1985-2017. **Supplemental Table 3.** Adjusted Hazard Ratios using Competing Cox Models for Incident Cardiovascular Diseases According to Incarceration Status, Overall and Stratified by Sex and Race, CARDIA Study. **Supplemental Table 4.** Baseline Characteristics According to Incarceration Status, Stratified by Sex and Race, CARDIA Study

## Data Availability

CARDIA complies with data sharing requirements of the National Institutes of Health by providing limited-access data sets from various CARDIA examinations to the National Heart, Lung and Blood Institute BioLINCC. Data are available through BioLINCC.

## Abbreviations

CARDIA: Coronary Artery Risk Development in Young Adults
CI: Confidence intervals
CVD: Cardiovascular disease
HR: Hazard ratio
