# Supplementary material for: Association between incarceration and incident cardiovascular disease events: results from the CARDIA cohort study
Source: BMC Public Health. 2021 Jan 26;21:214. doi: 10.1186/s12889-021-10237-6 (PMC7836455; doi:10.1186/s12889-021-10237-6)
Supplement: Supplementary file 1 — Additional file 1: Supplemental Figure. Participant Flowchart [file 12889_2021_10237_MOESM1_ESM.docx]

**Supplemental Figure.** Participant Flowchart.

Initial Sample:

5,115 CARDIA participants

Study Sample:

5,105 CARDIA participants

Reasons for excluding in study sample:

- Cardiovascular disease event prior to Year 2 examination (n=6)
- Transgender participant (n=2)
- No response to incarceration question (n=1)
- Withdrew consent (n=1)
