# Supplementary material for: Association between incarceration and incident cardiovascular disease events: results from the CARDIA cohort study
Source: BMC Public Health. 2021 Jan 26;21:214. doi: 10.1186/s12889-021-10237-6 (PMC7836455; doi:10.1186/s12889-021-10237-6)
Supplement: Supplementary file 2 — Additional file 2: Supplemental Table 1. Incarceration History by Exam Year, Stratified by Sex and Race, CARDIA Study. Supplemental Table 2. Unadjusted Event Rates for Incident Fatal and Non-fatal Cardiovascular Diseases and All-Cause Mortality According to Incarceration History Status, CARDIA Study: 1985-2017. Supplemental Table 3. Adjusted Hazard Ratios using Competing Cox Models for Incident Cardiovascular Diseases According to Incarceration Status, Overall and Stratified by Sex and Race, CARDIA Study. Supplemental Table 4. Baseline Characteristics According to Incarceration Status, Stratified by Sex and Race, CARDIA Study [file 12889_2021_10237_MOESM2_ESM.docx]

**SUPPLEMENTARY MATERIALS**

Supplemental Table 1. Incarceration History by Exam Year, Stratified by Sex and Race, CARDIA Study.

Supplemental Table 2. Unadjusted Event Rates for Incident Fatal and Non-fatal Cardiovascular Diseases and All-Cause Mortality According to Incarceration History Status, CARDIA Study: 1985-2017.

Supplemental Table 3. Adjusted Hazard Ratios using Competing Cox Models for Incident Cardiovascular Diseases According to Incarceration Status, Overall and Stratified by Sex and Race, CARDIA Study.

Supplemental Table 4. Baseline Characteristics According to Incarceration Status, Stratified by Sex and Race, CARDIA Study.

**Supplemental Table 1. Incarceration History by Exam Year, Stratified by Sex and Race, CARDIA Study.**

| **Group** | **Year 0 (Baseline)** | **Year 2** | **Both** | **None** |
| --- | --- | --- | --- | --- |
| White Male, n (%) | 30 (2.6) | 30 (2.6) | 12 (1.1) | 1,094 (93.7) |
| Black Male, n (%) | 82 (7.2) | 70 (6.1) | 43 (3.7) | 960 (83.1) |
| White Female, n (%) | 15 (1.2) | 9 (0.7) | 0 (0) | 1,281 (98.2) |
| Black Female, n (%) | 28 (1.9) | 27 (1.8) | 4 (0.3) | 1,419(96.0) |
| **Total, n (%)** | **155 (3.0)** | **136 (2.7)** | **60 (1.2)** | **4,754 (93.1)** |

**Supplemental Table 2. Unadjusted Event Rates for Incident Fatal and Non-fatal Cardiovascular Diseases and All-Cause Mortality According to Incarceration History Status, CARDIA Study: 1985-2017.**

|  | **Overall** | |  |
| --- | --- | --- | --- |
| **Event** | **Incarceration**  **(n=351)** | **No Incarceration**  **(n=4,754)** | ***P* Value** |
| Cardiovascular disease, No.* (%) | 33 (9.4) | 280 (5.9) | 0.008 |
| Coronary heart disease, No. (%) | 16 (4.6) | 142 (3.0) | 0.10 |
| Stroke, No. (%) | 6 (1.7) | 67 (1.6) | 0.28 |
| Heart failure, No. (%) | 12 (3.4) | 75 (1.6) | 0.01 |
| All-cause mortality, No. (%) | 53 (15.1) | 378 (8.0) | <0.0001 |

*Some participants had more than one event, which explains why the individual outcomes are greater than the total number of cardiovascular disease events.

**Supplemental Table 3. Adjusted Hazard Ratios using Competing Cox Models for Incident Cardiovascular Diseases According to Incarceration Status, Overall and Stratified by Sex and Race, CARDIA Study.**

| **Event** | **n/N** | **Model 1**  **HR (95% CI)** | **Model 2**  **aHR (95% CI)** | **Model 3**  **aHR (95% CI)** |
| --- | --- | --- | --- | --- |
| **Cardiovascular disease** | |  |  |  |
| Overall | 313/5105 | 1.63 (1.13-2.33) | 1.33 (0.91-1.95) | 1.28 (0.86-1.91) |
| White Men | 78/1167 | 2.30 (1.19-4.46) | 2.74 (1.38-5.42) | 2.36 (1.10-5.09) |
| Black Men | 102/1155 | 0.91 (0.54-1.56) | 0.96 (0.56-1.64) | 0.86 (0.48-1.54) |
| White Women | 38/1305 | 1.43 (0.20-10.28) | 1.48 (0.21-10.71) | 1.17 (0.12-11.10) |
| Black Women | 95/1478 | 1.66 (0.72-3.80) | 1.69 (0.74-3.89) | 1.56 (0.27-1.51) |
| **Non-cardiovascular death** | |  |  |  |
| Overall | 316/5105 | 1.90 (1.36-2.66) | 1.51 (1.07-2.14) | 1.15 (0.79-1.68) |
| White Men | 68/1167 | 2.05 (0.98-4.27) | 2.24 (1.07-4.67) | 1.44 (0.67-3.09) |
| Black Men | 123/1155 | 1.40 (0.90-2.15) | 1.44 (0.93-2.23) | 1.14 (0.68-1.92) |
| White Women | 50/1305 | --* | --* | --* |
| Black Women | 75/1478 | 1.19 (0.48-2.96) | 1.23 (0.49-3.07) | 0.96 (0.39-2.37) |

Model 1: Unadjusted.

Model 2: Adjusted for baseline age, sex, and race.

Model 3: Adjusted for baseline age, sex, race, baseline education, baseline smoking, baseline excessive alcohol use, baseline physical activity, baseline body mass index, baseline systolic blood pressure, baseline blood pressure lowering medication use, baseline total cholesterol, and baseline high-density lipoprotein cholesterol.

Cardiovascular events included fatal and non-fatal coronary heart disease, stroke, and heart failure events.

aHR: Adjusted hazard ratio. *Too few or no events for this outcome in the incarcerated group during follow-up in this sex/race group to produce stable modeled estimates.

|  | **White Men** | |  | **Black Men** | |  | **White Women** | |  | **Black Women** | |  |
| --- | --- | --- | --- | --- | --- | --- | --- | --- | --- | --- | --- | --- |
| **Baseline Characteristic** | **Incarceration (n= 73)** | **No Incarceration**  **(n= 1094)** | ***P***  **Value** | **Incarceration (n= 195)** | **No Incarceration**  **(n= 960)** | ***P* Value** | **Incarceration**  **(n= 24)** | **No Incarceration**  **(n= 1281)** | ***P* Value** | **Incarceration (n= 59)** | **No Incarceration**  **(n= 1419)** | ***P* Value** |
| Age, mean (SD), years | 24.2 (3.7) | 25.5 (3.3) | 0.001 | 23.8 (3.7) | 24.2 (3.8) | 0.12 | 24.7 (3.7) | 25.5 (3.4) | 0.26 | 24.3 (3.7) | 24.4 (3.9) | 0.79 |
| Education, mean (SD), years | 13.0 (2.1) | 14.7 (2.5) | <.0001 | 12.1 (1.4) | 13.1 (1.9) | <.0001 | 13.9 (2.7) | 14.6 (2.2) | 0.14 | 12.5 (1.8) | 13.1 (1.8) | 0.003 |
| SBP, mean (SD), mmHg | 114.0 (10.5) | 114.3 (10.2) | 0.77 | 115.7 (10.8) | 115.7 (10.6) | 0.99 | 104.9 (8.8) | 104.8 (9.4) | 0.95 | 106.7 (11.6) | 108.3 (10.0) | 0.24 |
| DBP, mean (SD), mmHg | 69.6 (9.9) | 70.9 (9.3) | 0.24 | 70.4 (10.4) | 70.6 (10.3) | 0.80 | 67.0 (8.9) | 66.1 (8.4) | 0.60 | 64.7 (12.2) | 67.6 (9.4) | 0.03 |
| BMI, mean (SD), kg/m^2^ | 23.6 (3.4) | 24.3 (3.6) | 0.07 | 24.0 (4.0) | 24.7 (4.4) | 0.05 | 23.1 (3.8) | 23.1 (4.4) | 0.95 | 25.3 (6.0) | 25.9 (6.6) | 0.50 |
| Total cholesterol, mean (SD) mg/dL | 176.8 (39.8) | 176.2 (33.4) | 0.87 | 177.0 (34.2) | 175.7 (35.3) | 0.64 | 167.6 (36.3) | 176.3 (30.9) | 0.17 | 171.7 (29.6) | 178.7 (34.2) | 0.12 |
| LDL, mean (SD), mg/dL | 109.2 (36.3) | 111.8 (30.5) | 0.49 | 105.9 (32.3) | 108.7 (32.8) | 0.27 | 99.8 (29.3) | 106.1 (28.8) | 0.29 | 105.6 (27.0) | 110.8 (32.3) | 0.22 |
| HDL, mean (SD), mg/dL | 51.0 (17.2) | 46.6 (10.5) | 0.001 | 56.1 (14.6) | 53.0 (13.6) | 0.004 | 52.3 (10.6) | 56.2 (13.1) | 0.14 | 52.1 (12.2) | 55.3 (12.9) | 0.06 |
| Triglycerides, median (IQR), mg/dL | 69 (51, 103) | 73 (52, 103) | 0.40 | 67 (48, 88) | 59 (44, 82) | 0.06 | 71.5 (44, 109) | 61 (45, 81) | 0.22 | 54 (45, 85) | 56 (42, 75) | 0.90 |
| Fasting plasma glucose, mean (SD) mg/dL | 86.9 (9.8) | 84.8 (10.8) | 0.12 | 83.2 (9.0) | 84.1 (17.5) | 0.48 | 82.4 (8.9) | 81.2 (13.5) | 0.67 | 80.1 (9.2) | 80.9 (21.6) | 0.78 |
| Heavy/moderate exercise, median (IQR), MET-minutes per week | 482  (303, 657) | 462  (287, 672) | 0.71 | 502  (298, 798) | 465  (264, 708) | 0.10 | 332  (253, 553) | 351  (205, 543) | 0.68 | 246  (183, 397) | 228  (102, 396) | 0.20 |
| Excessive alcohol, No. (%) | 27 (37.0) | 157 (14.5) | <.0001 | 55 (28.4) | 101 (10.6) | <.0001 | 7 (29.2) | 201 (15.7) | 0.09 | 9 (15.3) | 91 (6.4) | 0.02 |
| Individual income <$25,000, No. (%)* | 25 (41.0) | 263 (26.9) | 0.02 | 104 (68.9) | 317 (43.6) | <.0001 | 12 (54.6) | 293 (25.6) | 0.002 | 32 (69.6) | 589 (51.7) | 0.02 |
| Smoker, No. (%) | 37 (51.4) | 269 (23.2) | <.0001 | 110 (57.6) | 310 (32.7) | <.0001 | 11 (47.8) | 342 (26.8) | 0.02 | 32 (54.24) | 429 (30.4) | 0.0001 |
| Illicit drug use, No. (%)** | 15 (21.4) | 92 (9.0) | <.001 | 49 (28.0) | 88 (11.0) | <.0001 | 6 (25.0) | 68 (5.7) | <.001 | 16 (28.6) | 64 (5.2) | <.0001 |
| Blood pressure medication, No. (%) | 0 (0.0) | 19 (1.7) | 0.62 | 6 (3.1) | 19 (2.0) | 0.41 | 0 (0.0) | 14 (1.1) | 1.00 | 2 (3.4) | 55 (3.9) | 1.00 |
| Diabetes medication, No. (%) | 0 (0.0) | 2 (0.2) | 1.00 | 1 (0.5) | 1 (0.1) | 0.31 | 0 (0.0) | 5 (0.4) | 1.00 | 0 (0.0) | 5 (0.4) | 1.00 |

**Supplemental Table 4. Baseline Characteristics According to Incarceration Status, Stratified by Sex and Race, CARDIA Study.**

*Missingness ranged from 11% (white men group) to 24% (Black men group).

**Missingness ranged from 6% (white men group) to 16% (Black men group).
